# Exploring Community Stakeholders’ Perceptions of the Enhancing Family Well-being Project in Hong Kong: A Qualitative Study

**DOI:** 10.3389/fpubh.2017.00106

**Published:** 2017-05-15

**Authors:** Joanna T. W. Chu, Sophia S. Chan, Sunita M. Stewart, Qianling Zhou, Charles Sai-Cheong Leung, Alice Wan, Tai Hing Lam

**Affiliations:** ^1^School of Public Health, University of Hong Kong, Hong Kong, China; ^2^School of Nursing, University of Hong Kong, Hong Kong, China; ^3^Department of Psychiatry, University of Texas Southwestern Medical Center at Dallas, Dallas, TX, USA; ^4^Information Systems and Technology Branch, Social Welfare Department, Hong Kong, China

**Keywords:** qualitative, community engagement, community stakeholders, community-based interventions, family intervention

## Abstract

**Background:**

Community engagement is a powerful tool in bringing about positive social and community change. Community stakeholders possess critical experience and knowledge that are needed to inform the development of community-based projects. However, limited literature is available on the practical experience involved with planning and implementing community-based family programs. Even less has been published documenting efforts in Chinese communities. This paper explores community stakeholders’ experiences with the enhancing family well-being project—part of a citywide project entitled the “FAMILY Project,” aimed at promoting family health, happiness, and harmony in Hong Kong.

**Methods:**

This qualitative evaluation examined the perspectives of community stakeholders. Four focus groups with social workers (*n* = 24) and six in-depth interviews with steering committee members were conducted from December 2012 to May 2013 in Hong Kong. Focus groups and in-depths interview were audiotaped, transcribed, and analyzed using thematic analysis techniques.

**Results:**

Rich accounts were given by our respondents on various aspects of the project. Main themes and subthemes were identified and grouped into four categories (project conception, project implementation, project consolidation, and the overall impact of the project). Respondents described the practical challenges associated with the project (e.g., recruitment, balancing scientific research, and lack of resources) and identified the elements that are important to the success of the project. These included the commitment to a shared goal, multi-agency collaboration, and a platform for knowledge exchange. Finally, respondents perceived benefits of the project at both the individual and community level.

**Conclusion:**

Our project sheds light on many of the practical considerations and challenges associated with a designing and implementing a community-based family intervention project. Community stakeholders input provided important information on their perceived benefits and barriers and can inform and improve future development of community-based family intervention programs.

## Introduction

Family is a vital component of any society. Well-functioning families contribute to the stability and well-being of society (1). Supporting and addressing the needs of families continues to be the focus of many researchers and policy makers (e.g., National Research Council and Institute of Medicine). In view of this, The FAMILY Project (FAMILY: a Jockey Club Initiative for a Harmonious Society—https://www.family.org.hk), funded by The Hong Kong Jockey Club Charities Trust and in collaboration with the School of Public Health of the University of Hong Kong was established to enhance three outcomes salient to Hong Kong families: health, happiness, and harmony. In line with a number of international bodies (e.g., Institute of Medicine and Centers for Disease Control and Prevention) that have strongly advocated for community engagement and community-based research efforts, The FAMILY Project actively engaged the community in collaboration research and community capacity building. The objective of these efforts was to combine knowledge and action of community partners and academics to improve community health and reduce disparities (2). Community engagement offers numerous benefits including increased relevance of the science to local community partners; empowerment of the community’s ability to vocalize and address its needs, and the use of the community’s strengths and resources to initiate and conduct research (3). For this paper, community engagement refers to community involvement in decision-making and in the design and delivery of initiatives that promote and improve family well-being.

Despite the numerous advantages of community engagement, such an approach is understudied in non-Western societies. Very little is known about the practical experience involved with community-based family interventions. The success of engagement and interventions implemented at the community level depends significantly on the receptivity and commitment to community projects among stakeholders (4). For the purpose of this paper, community stakeholders are defined as those involved in project operation (i.e., social workers and steering committee members—consisting of both frontline workers and those who may play administrative roles in their organizations). One recommended approach to community engagement is the use of a “participatory approach” (5). This includes involving the relevant stakeholders in identifying problems and contributing to solutions. Evaluations are commonly used to gain insight to program implementation, to improve the intervention projects, and to determine whether the impacts and effects are achieved (6). The extent to which the community-based family interventions are seen as relevant and feasible is an important aspect in predicting program uptake and long-term sustainability (7). The practicalities of implementing community-based interventions and a clearer understanding of real-world issues are both necessary for policy planning, evidence-based decision-making, and for future practice development (8).

The aim of this paper, utilizing a qualitative approach, is, therefore, to describe the experiences and perspectives of community stakeholders on the enhancing family well-being project—a part of the FAMILY project and evaluate the success and challenges utilizing a community-based research model. The perspective of those served (i.e., families) have been reported elsewhere.

### Enhancing Family Well-being Project

The Sham Shui Po district is the fourth most densely populated local district in Hong Kong. The proportions of single parents, elderly, new immigrates from mainland China, ethnic minorities, and comprehensive social security assistance recipients in this district are relatively high. Of concern in recent years is the increasing trend in various family problems, such as, divorce, extramarital affairs, abuse and neglect of children, and domestic violence. To tackle these family problems, the enhancing family well-being project was developed collaboratively between Social Welfare Department of Sham Shui Po District Office and the FAMILY project team of the School of Public Health, the University of Hong Kong. The aim of the project was to raise public awareness around the importance of family communication and to enhance family relationships and family well-being. Importantly, we sought to bridge the gap between knowledge produced through research and translation into real-world practice. Based on a community-based research model that perceives community as resource, our project focused on training and mobilizing local community stakeholders, and developing, implementing, and evaluating community-based interventions for families. We believe that a high degree of community ownership and participation is essential for sustained success. To maximize participation and community engagement, a steering committee consisting of representatives from various community partners was formed to oversee and provide supervision of the project.

The project was carried out in three stages: project conception, project implementation, and project consolidation. The first stage involved a launching ceremony to publicize and raise awareness about the project. Members of the public were invited to complete a short questionnaire reporting on their family well-being on the day of the launching ceremony. The preliminary data were used to identify the needs of families and in turn better tailor future interventions that would be relevant and culturally appropriate to families. Capacity building train-the-trainer programs were organized by academic members and community partners to equip participating social workers with relevant knowledge and skills for translating the project aims into effective intervention programs (9). Each participating NGO assigned one to two social workers (the typical frontline service delivery professional in Hong Kong community service agencies) from their organization to participate in the training program. The social workers were expected to deliver interventions to their targeted clients.

The second stage involved the implementation of community-based family interventions. Based on a positive psychology framework that was conveyed during the training program, participating NGOs designed family interventions to meet the needs of their service clients. To maximize the quality and consistency of the interventions across NGOs, standard guidelines and protocols were provided on program design. Each participating NGO submitted individual program proposals to the project steering committee for feedback and funding approval before program implementation. Families were then recruited by the NGOs from both their active clientele and residents of the community to participate in the community-based interventions. A total of 1,734 participants from 1,069 families participated in the interventions. A detailed report on the intervention trial including its design and effectiveness is presented elsewhere (9).

The final stage, project consolidation involved activities organized to disseminate preliminary findings of the project to the community stakeholders and members of the public. Community stakeholders were invited to share their experience in a practice wisdom forum on taking part in the project, and to discuss project outcomes. An education booklet, which contained strategies for enhancing family relationship as well as several case studies from families who participated in the project, was distributed to members of the public. A practice manual that included the project rationale, theoretical framework, training materials, as well as practical experience gained from program planning and implementation, was distributed to community stakeholders for knowledge transfer and consolidation.

Throughout the project, the academic members were primary contributors to the theoretical aspects and to the science of the project (i.e., methodology, development of assessment and quality control materials, data management and analysis), while NGO partners contributed their professional experience with the needs of the local community (i.e., design and implementation of interventions, recruitment, and data collection). Communication between and within academic members and community stakeholders was frequent, with quarterly progress reports from NGOs shared among project staff.

## Materials and Methods

The use of qualitative methods in community-based evaluation has been recognized as a vital part of the decision-making processes assisting with policy and practice development (10). Thus, focus groups and in-depth interviews were utilized to provide a rich understanding of the project from the perspective of the community agency frontline workers who implemented it.

### Participants

Purposive sampling was used. Email invitations were sent to all participating NGOs (*n* = 30) inviting their frontline workers who were involved in the delivery of the interventions to participate in focus groups. Interested participants contacted our research team and information or any questions were addressed at this point. Participant information sheet and a letter confirming the date, time, and location of the focus group were then sent to eligible participants. The focus groups and in-depth interviews were conducted at local organizations to provide ease of access. A total of four focus groups (*n* = 24 social workers, representing 21 participating NGOs) were conducted in December 2012, following project implementation. The number in each group ranged from 5 to 7. Respondents were mostly woman, with a mean age of 32.5 years. More than half had worked in the existing participating units for over 5 years.

All members of the steering committee (*n* = 9) were invited to participate in in-depth interviews. A total of six in-depth interviews were conducted between March and May 2013, following the project consolidation stage. Participants were recruited to represent the diverse range of organizations involved in the project (i.e., District Office, Education Bureau, The Boys’ and Girls’ Clubs Association of Hong Kong, Sham Shui Po District Social Welfare Office of Social Welfare Department, Hong Kong Family Welfare Society, and the Neighborhood Advice-Action Council). Half of the respondents were females, with the majority aged between 45 and 54 years. All had acquired tertiary education. Two-thirds had worked in the participating unit for 10 years or above.

### Procedures

Participation in the focus group discussions or individual interviews was voluntary, and written informed consent (and a questionnaire on demographics) was collected from the participants before the discussion began. Semi-structured interview guidelines and prompts were developed based on Krueger and Casey (11) questioning technique to cover a range of key issues related to the research questions and used in focus groups and in-depth interviews. Questions focused on the experience of stakeholders on various stages of the project including project conception (i.e., What were your thoughts on the conception stage of the project? What did you find helpful? What did you find least helpful?), implementation (What was your general impression of implementing and delivering the intervention? What worked? What didn’t work? How were these resolved?), and consolidation (What was your general impression of the consolidation stage? What did you find helpful? What was least helpful?). The overall impact of the project at both the individual and community level and recommendations were also explored. Prompts were used to ensure coverage of the key issues related to the research questions. The focus groups and interviews were conducted with flexibility to allow unanticipated themes to emerge. The focus group lasted approximately 90 min, and the interview lasted for approximately 60 min.

### Data Analysis

All focus groups and in-depth interviews were audiotaped and transcribed verbatim into Cantonese. At least 10% of the transcripts were double-checked against the tape recordings. In-depth interviews and focus groups data were combined for the purposes of data completeness and confirmation (12). To ensure data integration of focus groups and in-depth interviews, the research team moved back and forth between the data sets to discover data convergence, divergence, and complementarity (12). Thematic analysis (13) was used to identify, analyze, and report patterns (themes) within the two data sets from the study. The predetermined themes were formed from the key research questions, which included the three project stages and the overall impact of the project. Data from focus groups and interviews were amalgamated. At the beginning, the transcripts were read in detail, and broad themes were noted. Then, an in-depth analysis was conducted using a process of constant comparisons, in which differences and similarities were analyzed to identify main themes and sub-themes. Triangulation of data was achieved by three independent researchers approaching the same sources and independently coding the focus groups and in-depth interviews, reduced the potential for researcher bias. This revealed that, in 92% of cases, the same codes were given to the same excerpts, which led to further refinement of the definitions of each main and subtheme, as well as the content coded under each theme, where necessary. An audit trail was kept throughout the process of data collection and analysis, and included field, process, and reflexive notes. Peer debriefing was used and involved ongoing discussion between researchers during the course of focus groups. Finally, informal member checking occurred during data collection when the moderator reflected back his/her understanding of participant responses and sought feedback regarding whether this understanding was accurate.

## Results

The findings were organized into four main categories. These included themes identified in relation to various stages of the project: (1) project conception, (2) project implementation, (3) project consolidation, and finally, (4) the overall impact of the project. These themes were illustrated with quotations. The quotations were taken from a number of respondents and are identified based on whether they were obtained from members of the steering committee (C) or social workers (S). For the latter, identification also includes the focus group (G) to which the social worker belonged.

### Stage 1: Project Conception

As part of the project conception stage, capacity training programs were provided by academic staff to community stakeholders. Many respondents felt the training program was comprehensive and equipped them with skills that were relevant and applicable to their work.

I was impressed by this part (training program). It is uncommon for projects to include such comprehensive pre-program preparation and training. Relatively speaking, our colleagues were clear about what they had to do (G1, S3).

#### Requesting for More In-depth Training

The need for more training was expressed by respondents. Suggestions were made for the content of the training, such as more in-depth positive psychology training, as well as the format of the training (i.e., longer discussion time).

Continuous training is needed … colleagues need mutual support at different stages (of the project). Mutual support can be gained through sharing of experiences and acquiring new knowledge and work skills (C6).

### Stage 2: Project Implementation

The second stage of the project involved the design and implementation of interventions by community stakeholders. Respondents were asked about their experiences with the roll out of the interventions. Balancing scientific research, recruitment, and the lack of resources were prominent themes that emerged.

#### Balancing Scientific Research

Community partners acknowledged the difficulty in balancing the needs of the community and research rigor. Some respondents felt that the project maintained strong scientific standards more than it accommodated the needs of the community. The challenge with completing questionnaires (e.g., difficult wording, long and repetitive, and difficult to follow-up with participants) was repeatedly noted by respondents.

When you (academic staff) plan a research project, there are a lot of practical issues that can be difficult to do in the community. I think a lot of our staff struggled with the questionnaires, the fixed duration time of the intervention, and recruitment. It imposes a lot of restrictions on our frontline workers (G3, S13).

#### Recruitment

Issues relating to recruitment were a recurring theme throughout the focus groups and in-depth interviews. A number of respondents described their recruitment strategies. Initial recruitment focused on those who were already working with families, often those with higher level support needs in relation to family functioning. Social workers described engagement with families prior to their attendance as a key part of the recruitment process. Many social workers noted that a proactive approach was needed in order for recruitment to be successful. Schools and churches were identified as being one way to help disseminate information about the community-based programs. This included distributing leaflets and encouraging school/church staff to promote the interventions and/or make referrals themselves.

We used a lot of different methods (to recruit). We had banners, distributed leaflets, and our colleagues called the participants individually (G4, S22).

We did collaborations with schools. Through schools, we recruited more families that were suitable and within our target group (C5).

Despite a number of recruitment strategies used, social workers frequently stated that recruitment of families was a challenging issue. Many commented that families did not have the time to participate in interventions. The interventions were also not considered a priority compared with the other commitments or problems that families had to deal with. Specific sub-groups of individuals, such as male and single-parent households were identified by respondents as particularly challenging to recruit.

(Low male participation) was not related to the design of the project; it’s actually a universal issue. I think there is a universal need to come up with methods to involve more fathers or male participants in our community projects (C6).

It’s difficult for a “broken” family to participate, but they are the ones that we really want to reach. They are more disadvantaged, but we (the project) did not really captured or reached them (C2).

Moreover, recruitment was deemed difficult due to the number of NGOs competing for participants in the same district and at similar time points.

There were 30 organizations in the district that participated in the project. Did we really have so many families to recruit? (G2, S8).

#### Lack of Resources

Issues related to resources were identified as major challenges for organizations to overcome during the implementation stage. Although each participating NGO received funding, respondents felt that the amount was insufficient to cover all the cost required to implement the interventions. Almost all respondents noted that the project had taken substantial time and effort to complete. Developing and implementing the interventions, recruitment of participants, training of volunteers, and completing paperwork added a significant layer of demand to the workload of respondents.

Generally speaking, there were many technical and resources issues. There was not a lot of support from the Sham Shui Po district. I think a lot of the organizations (involved) had to put in their own money and manpower to participate in the project (G4, S26).

Mixed responses were reported by respondents on the level of support received from university staff. Some respondents noted that they felt a lot of the support was given at the beginning stage of the project (i.e., training and designing intervention), however, university staff were less invested during the program implementation stage. On the other hand, some respondents cited the involvement of academic staff was a strength for the delivery of interventions. University staff members were present and available throughout the roll-out process, supplied service providers with a detailed overview of the project, project materials, and oversaw all phases of the implementation.

I think there could have been better communication (with university staff). It would be better if we dealt with the same person each time, so they were more familiar with what we did in the last session and knew how to deal with the problems we encountered previously (G2, S12).

(Academic staff) followed throughout the project and gave us a lot of support. The knowledge they have was a huge back up for us (C4).

### Stage 3: Project Consolidation

Members of the steering committee were highly appreciative of the practice wisdom forum that allowed community stakeholders to discuss and reflect upon the effectiveness of the interventions and the overall project. Respondents further noted that the dissemination of the findings from academic staff was useful and could help with future practice and development.

There were around a thousand families that participated in the project. The rich set of data can help us to further promote our services and strengthen the implementation of programs in the district. I think we have gained a lot (C4).

The academic team presented the science and data, and these data helped us as front line workers to promote, enhance, and improve our service (C3).

### Overall Impact

Rather than focusing on the effectiveness of the community-based interventions [reported elsewhere (9)], we examined the overall impact of the project. The overall response to being involved in the project was positive, with many perceiving the project as successful. All respondents identified benefits in collaborating with multiple agencies and forming community–academic partnerships. These benefits included investment in a shared goal, interaction across agencies, and exchange of knowledge.

#### Investment in a Shared Goal

Elements used to characterize a positive collaborative experience were the emphases and existence of a shared goal to all involved. Respondents felt that the project was beneficial to their community and were therefore more willing to invest and commit to the project.

We had various NGOs serving various clients. I think the fact that we share a common goal, we were able to learn together, write proposals together, implement together, and draw a conclusion together (C8).

#### Interaction across Agencies

Community stakeholders reported feeling included and valued the opportunity in being a part of the project. Respondents repeatedly stated that the project was a big initiative in their community.

The project further brought together organizations with different services and backgrounds, mutual understanding and communication was enhanced. This is what we were looking for before (the project) (C4).

We collaborated, and all of us devoted ourselves into the project. It was because of everyone, that this project was successful. We couldn’t have done it by ourselves (G3, S16).

#### Knowledge Exchange

Respondents noted that the collaboration and involvement in the project created avenues for them to learn, exchange knowledge, and share their areas of expertise.

Our colleagues gained a lot from acting as frontline workers (by receiving) valuable information, training, articles, wisdom sharing, and exhibitions. There was a lot of communication that enriched their way of thinking (C4).

The success of the project depended significantly on forming effective partnerships. Three salient factors were raised in relation to the development and maintenance of partnerships: building trust, problem solving, and open communication.

#### Building Trust and Relationship

Establishing and building trust was essential for successful partnership. Respondents felt that a lot of trust and openness existed within and between partners since the project started. Many respondents reported feeling very respected by others.

The working relationships between and within organizations, university, and government departments have become stronger. While we were collaborating, we got to know each other, we expressed our views and we were able to understand and discuss various views and problems openly. This (project) created a platform for future collaboration (C4).

We worked with various NGOs and different organizations (i.e., university, Social Welfare Department) for more than 10 months. We have become very close-knit. We built our working relationship right from the beginning all the way to the end (C8).

#### Problem Solving

While there was an implicit acceptance and support for working with multiple NGOs and community partners, it was apparent that with many NGOs, differences in perspectives sometimes led to difficulties in coordination and communication. Being able to problem solve and finding common ground within and between partners led to an enhanced collaborative experience.

The most challenging part was to bring together the 30 organizations in the district. Each organization has its own characteristics, work and organization culture, not to mention its own ways of implementing the programs. It is about how organizations of different nature (work together) and about how to arrange for social workers to follow the same procedures and convey the same messages to service targets (C3).

#### Open Communication

At the core of successful collaboration and building effective partnerships was fostering open communication. Maintaining regular, ongoing communication, between academic staff and NGOs, and soliciting their feedback reassured that the staff were invested and committed to the project and helping each provider succeed.

If you don’t communicate right from the beginning, then miscommunication can occur … we have 30 NGOs, it actually required a lot of communication, and open communication is key (C3).

#### Individual Impact

In addition to describing their overall experience with the project, respondents were further asked about the impact of the project on themselves. The majority of the respondents experienced positive benefits in terms of self-confidence and building social relationships. Obtaining confirmation through the quantitative assessments from families that their work was effective and that they were attaining their goals, was validating for all respondents.

I learnt a lot from this project…from organizing to planning and delivering the interventions was all a learning process, like how to engage and motivate participants. I learnt from them (participant) too, and realize that I can use positive psychology in the future with other people. It’s encouraging (G3, S16).

Despite the positive benefits, some social workers spoke of negative effects, such as exhaustion and stress. Respondents noted that they were often extremely busy, working under high pressure with limited time and staffing resources. Attending the training, designing, recruiting, and the delivery of the interventions were a potential drain on their limited resources of time and energy.

It was difficult for me to implement, to plan, to supervise, and to complete administrative work. It was hard for me to devote all my effort just on this project (G1, S2).

#### Community Impact

Respondents were also asked to comment on the potential impact of the project on the community. Positive comments were made by respondents including extended reach and an overall sense of positivity in the community.

Although there have been a lot of collaborative projects in Sham Shui Po district before, I think this project had the largest coverage and was the most in-depth (C6).

When there are many social problems that could not be solved at this moment (this project) that promoted well-being, which motivated people to live positively, is meaningful to society. We could not change the problem of poverty in a short time, but (the project) changed people’s attitude toward life and problems and people were happier (C1).

While many noted the positive impact of the project, others felt much more work is needed and emphasized that there is no “quick fix” to problems faced by families in the community.

Some deep-rooted issues can’t be solved by one or two programs (C2).

This is long-term work and the difficulty is that the effects might not be observable (C4).

Lack of continuity in community–academic partnership, together with the failure to embed projects into NGOs due to the short-term nature of resources and funding available, were noted as a challenge to long-term sustainability of project effects.

(The project) was meaningful, so we allocated additional resources (on top of funding). But if we wanted to sustain it, then where does the funding come from? When we want to promote these sort of programs, we can’t do it without funding. Staffing is also an issue. It’s a challenge to figure out how to use what we have to make an impact (C7).

Nonetheless, respondents were optimistic and believed that the project formed a solid foundation for their future work in the community.

(Through this project) we worked closely together, we used the same strategies, we shared the same vision, and we all had one goal. So if we look at it in the long term, this will slowly sink into our community (C4).

The knowledge (gained) can impact our future work and we can build upon it. It’s like you have planted a seed, and if we can continue then over the years, it will grow. So it’s a process (C3).

Members in the steering committee also spoke of the implications the project has for government policies. Respondents noted that the project was a good example for what effective collaboration among organizations can achieve in a community.

I think the project is rather successful and the policy makers could see it really worked. When a project theme meets the needs of the community and is accepted by the community, people from different sectors get involved to deal with or face the problems in the community. I think this project has set a good example (C4).

If you ask me whether this project might have made a long-term impact on policy making, the answer is yes. I saw a positive effect on the development of governmental staff. I think the project brought benefits to the community, and it may have an impact on long-term policies (C6).

## Discussion

An important goal of this qualitative evaluation was to understand community stakeholders’ practical experiences in a community-based research project and evaluate the success and challenges utilizing a community-based research model. The scarcity of published papers on qualitative data in this area, particularly in a non-Western setting, make our findings an important contribution to the literature.

Our enhancing family well-being project shed light on many of the successful elements and practical challenges in implementing a community-based family intervention project at the community level. The project brought together various organizations who followed a common goal that was relevant and culturally appropriate to the needs of the community. Chinese individuals are socially and culturally embedded in their broader Chinese community, working with broader community to design, recruit, and implement our community-based family intervention project was essential. Our community stakeholders were engaged from initiation at the project conception stage including framing the research goal. For example, although the general agenda of enhancing family well-being was set at the outset, there was considerable leeway with regards to how this agenda was met in terms of the structure of the program, the specific targets, and the strategies used to impact the targets. In addition, the community partners contributed their considerable expertise with regard to program implementation for their constituents including needs of families and recruitment. They were partners in decision-making regarding the feasibility of the timeline, the supports needed to minimize barriers to attendance, and problem solved as unexpected issues developed. Our respondents appreciated the diversity of the partners and the fact that so many organizations contributed to the project. There was overall a positive sense of the way in which various organizations worked together, particularly in terms of building trust and relationships, problem solving, and fostering open communication. This form of partnership between researchers and communities, with community participation contributing to the success of community programmes, has been described in the literature (14). Building relationships and cultivating positive connections in the Chinese community is important.

The practical challenges in relation to implementation and sustainability are consistent with those found in International literature on community-based projects (15–17). The challenges with balancing scientific research, recruitment, and the lack of resources suggest that programs delivered in the real world require resources on a variety of fronts, especially efforts to strengthen the infrastructure of agency practice. Recognizing that recruitment is difficult is needed and often there is an under-estimation of the effort required. Community partners are faced with competing demands and it is unrealistic to expect community partners to devote all their time and effort into recruitment, particularly for participants beyond their usual service targets. Community organizations’ capacity and readiness to organize themselves to undertake coordinated action are important issues to assess and consider prior to participation in research projects (3).

By obtaining the perspectives of community stakeholders, we have attempted to increase community acceptance and participation in research, improve data collection and intervention implementation, and enhance the capacity of various organizations. Building on the rich accounts from community stakeholders and lessons learnt from this project, we recommend the following for those interested in engaging communities in community-based research. First, a common set of goals and objectives need to be identified among partners. These goals and objectives should translate into specific, tangible actions that bring measurable benefits to the community. A key objective is to incorporate local knowledge into the project’s decision-making process and identify the needs of the community, thus leading to better tailored projects, better targeted outcome, and a sense of ownership among stakeholders (18).

Second, building trust among community partners is an ongoing process. Maintaining equitable partnerships is often difficult (3). Researchers cannot take for granted the responsibility of maintaining communication throughout the entire collaborative process. Building relationships among and within organizations is particularly important since relationships can develop into lasting partnerships leading to additional collaborations. For example, our FAMILY Project has, over the years, developed long-lasting relationships with various organizations that have served as the foundation for ongoing projects (2).

Third, stakeholder involvement in a community-based research project requires substantial time and effort. Community stakeholders are faced with competing demands and it is unrealistic to expect stakeholders to devote all their time and effort into a project. This suggests the importance of flexible and alternative methods of involving community partners and making realistic projections of time commitments. Clear expectations should be discussed up front.

Finally, the biggest challenge to community-based research project is often the funding required. Community organizations frequently lack the financial resources that create barriers to participation in community projects and may create tension and stress within and among organizations. Securing funding to ensure self-sufficiency and integrating interventions into the community proves to be difficult and is ultimately a matter of policy change.

One of the common limitations with qualitative research is the relatively small number of participants representing the project and the extent to which these findings can be applied beyond the specific group of individuals involved in the focus groups and in-depth interviews. Given the diversity within Asian ethnic groups, whether our experiences could be generalized is uncertain. However, improving understanding in one population may help raise awareness of possible recruitment issues for other community-based research. In addition, while we are able to demonstrate the benefits and potential of our project (e.g., community engagement, empowerment, and capacity building), we cannot appraise the lasting impact on the community.

## Conclusion

Notwithstanding the limitations, the rich set of responses from community stakeholders highlight the importance of obtaining community feedback on collaborative research in the community. Future projects should consider a community participatory approach to maximize the use of the expertise that various participating parties contribute. Our findings have important implications not only for future projects but also for family service policy planning at the government level.

## Ethics Statement

Ethics approval was obtained from the Institutional Review Board of the University of Hong Kong/Hospital Authority Hong Kong West Cluster.

## Author Contributions

The first author JC led the development of this manuscript. SC, QZ, AW, CL, and TL contributed to the research design. SC and ZQ helped to collect data. JC analyzed the data. SS made continual input as the draft progressed and approved the final draft for submission. TL and ZQ critically reviewed the final draft. All authors read and approved the final manuscript.

## Conflict of Interest Statement

The authors declare that the research was conducted in the absence of any commercial or financial relationships that could be construed as a potential conflict of interest.

## Abbreviations

NGO, non-government organization.
